# Synthesis and Structural Determination of Novel 5-Arylidene-3-*N*(2-alkyloxyaryl)-2-thioxothiazolidin-4-ones

**DOI:** 10.3390/molecules17033501

**Published:** 2012-03-19

**Authors:** Khaled Toubal, Ayada Djafri, Abdelkader Chouaih, Abdou Talbi

**Affiliations:** 1 Laboratoire de Synthèse Organique Appliquée, BP 1524, El Menaouer, Université d’Oran Es-Senia, 31000 Oran, Algérie; 2 Laboratoire de Structure, Elaboration et Applications des Matériaux Moléculaires (SEA2M), Département de Génie des Procédés, Faculté des Sciences et de la Technologie, Université de Mostaganem, 27000 Mostaganem, Algérie; Email: aek_chouaih@yahoo.fr

**Keywords:** *N*-arylthiazolidin-4-one, (*Z*)-5-arylidene-3-*N*-arylthiazolidin-4-one, chirality, IR, ^1^H-NMR, ^13^C-NMR, X-ray diffraction, nonlinear optical properties, solarcells

## Abstract

As part of our project devoted to the synthesis of heterocycles including thiazole rings, some new 5-arylidene-2-thioxo-3-*N*-arylthiazolidin-4-ones were synthesized by Knoevenagel condensation. An interesting feature of these compounds is that their chirality is induced by that of their 3-*N*-(2-alkyloxyaryl)-2-thioxothiazolidin-4-one precursors, which in turn is due to the presence of a C2 axis of chirality. These new compounds were characterized by spectroscopic methods (IR, ^1^H-NMR, ^13^C-NMR). The structure of compound (***Z***)-(**2g**) was further determined by X-ray diffraction.

## 1. Introduction

The increasing diversity of azolidinone heterocyclica, particularly thiazolidin-4 one derivatives [1,2,3,4,5] has been widely investigated for a range of pharmacological activities [6], such antiviral, anticonvulsant [6], antibacterial [7], hypolipidaemic and anti-inflammatory effects, and potential anticancer drug candidates [8,9,10]. In recent years, the push-pull effects of thiazolidinone derivatives have been receiving special attention of physicists and chemists for their nonlinear optical properties [11]. Previously, it was shown that the presence and the nature of the moiety in position 5 of thiazolidinones plays a key role in their biological and physical properties [11,12]. In continuation of our previous work on the development of organic photovoltaic cells [13], where 5-arylidene-3-*N*-arylthiazolidin-4-ones seems to be good candidate to fulfill our objectives, we focus now on the synthesis and structure elucidation of some new arylidenethiazolinones.

**Scheme 1:**
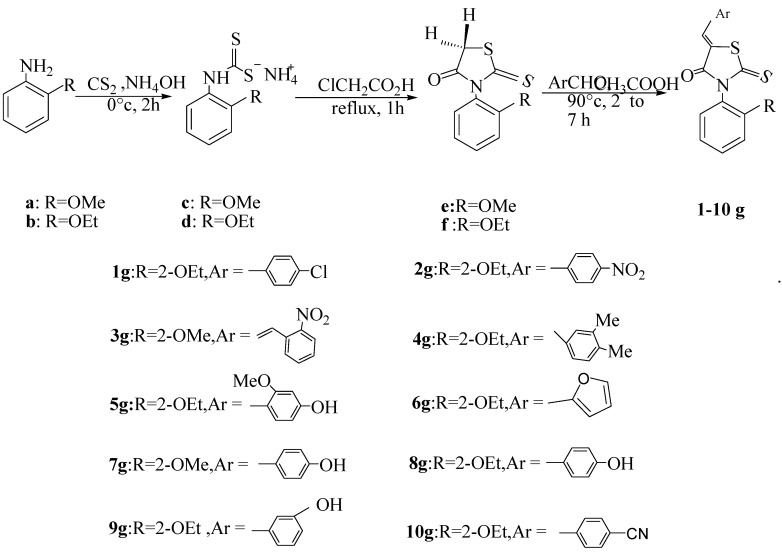
Synthesis of 5-arylidene-3-(2-alkyloxyaryl)-2-thioxothiazolidin-4-ones.

The syntheses of 5-arylidene-*N*-arylthiazolidin-4-ones have been carried out using a three steps sequence as follows (Scheme 1): (1) the reaction between carbon disulfide, aromatic amine **a,b** and ammonium hydroxide gives ammonium *O*-aryldithiocarbamate salts (DTC) **c,d**; (2) reaction between the DTCs and chloroacetic acid leads to *N*-arylthiazolidinones **e,f** [14]; (3) the *N*-arylthiazolidinones react with aromatic aldehydes to yield 5-arylidene-3-*N*-arylthiazolidin-4-ones **1g–10g**. The structures of these new compounds have been determined by different spectroscopic methods (IR, ^1^H-NMR and ^13^C-NMR, X-Ray diffraction).

## 2. Results and Discussion

**Figure 1:**
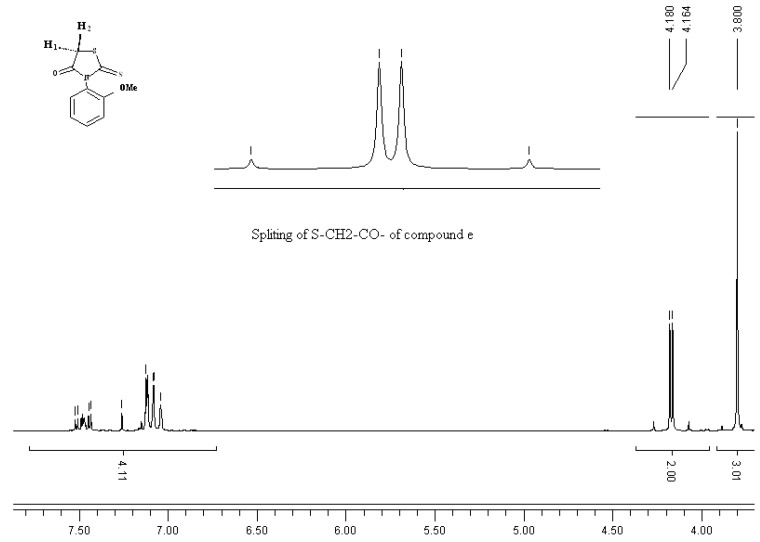
^1^H-NMR spectrum of 2-thioxo,-3-*N*(2-methoxyphenyl)thiazolidin-4-one **e**.

The IR spectra of all compounds showed *N*-arylthiazolidinone C=O stretch bands at 1694–1753 cm^−1^. In the ^1^H-NMR spectra of the *N*-arylthiazolidinone compounds, the diastereotopic protons H1 and H2 appear as a doublet of doublets (AB system) in the region of 4.07–4.27 ppm (J_1–2_ = 18.11 Hz) (Figure 1).

**Figure 2:**
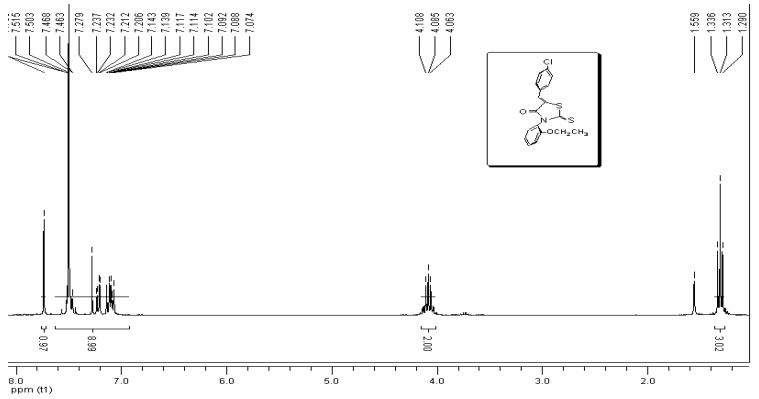
^1^H-NMR spectrum of (*Z*)-5-(4-chlorobenzylidene)-3-*N*(2-ethoxyphenyl)-2-thioxothiazolidin-4-one (**1g**).

**Figure 3:**
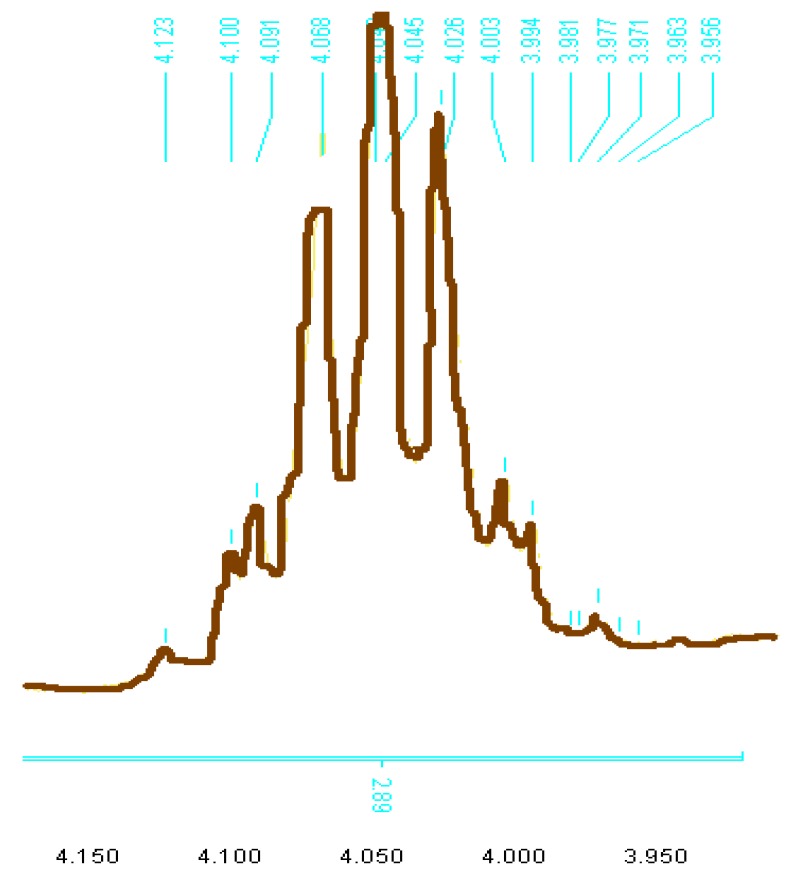
Splitting of O-CH2-CH_3_ of compound **8g**.

In the ^1^H-NMR spectra of all compounds containing 2-ethoxylphenyl substituents the geminal protons Ha and Hb appear as two octets (ABX_3_ system) in the 4.00–4.16 ppm region with a weak coupling constant (J_ab_ = 2.2 Hz), as shown in Figure 2 and Figure 3 [13,15]. These diastereotopic protons also contribute to the detection of axial chirality around the (Nhet-Caryl) bond (Figure 2). Aromatic proton signals appear in the 6.93–8.53 ppm range.

**Figure 4:**
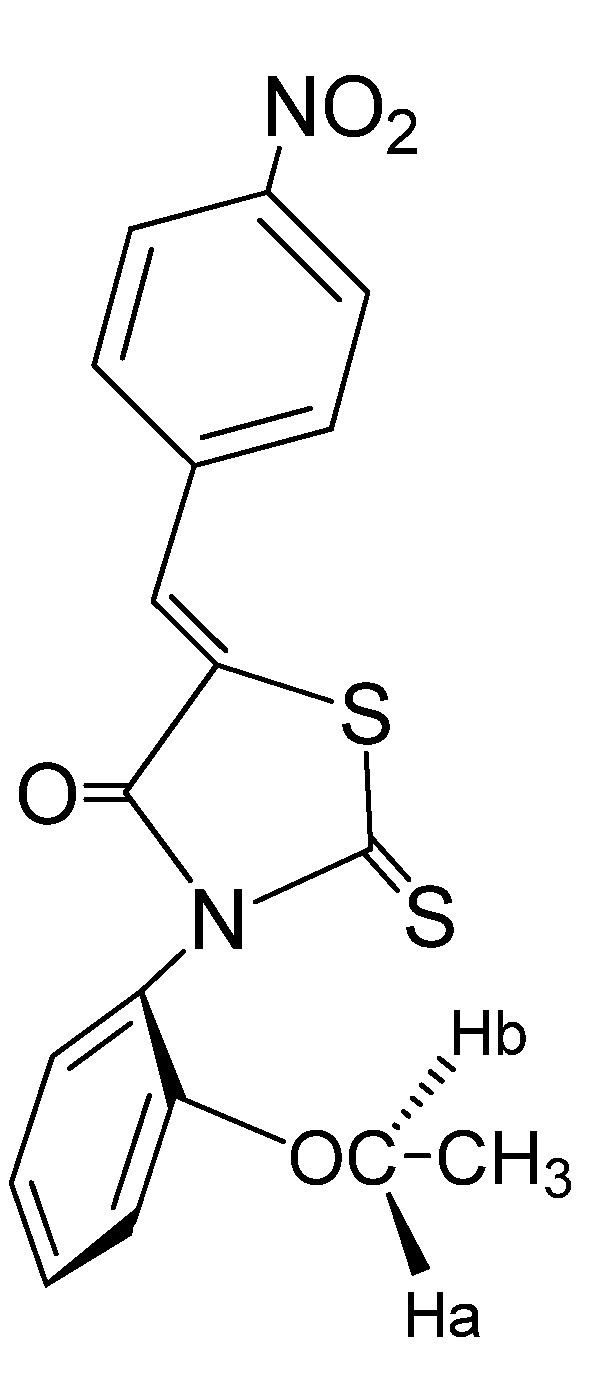
Structure of (*Z*)-5-(4-nitrobenzylidene)-3-*N*-(2-ethoxyphenyl)-2-thioxothiazolidin-4-one (**2g**).

In the IR spectra, the emergence of a broad band at 3,496 cm^−1^ highlights the presence of a hydrogen bond due to the electronic interaction between the carbonyl and the methine proton. This interaction can occur only in a *Z*-geometry. The (*Z*)-configuration of the exocyclic C=C bond in the 5-arylidene derivatives **1g–10g** was confirmed by the signal of a methine proton which resonated at higher chemical shift (7.51–8.03 ppm) as a singlet. The presence of this configuration is already reported in the literature [14]. We have further confirmed this fact by obtaining the X-Ray diffraction spectrum of compound **2g** (Figure 4).

**Figure 5:**
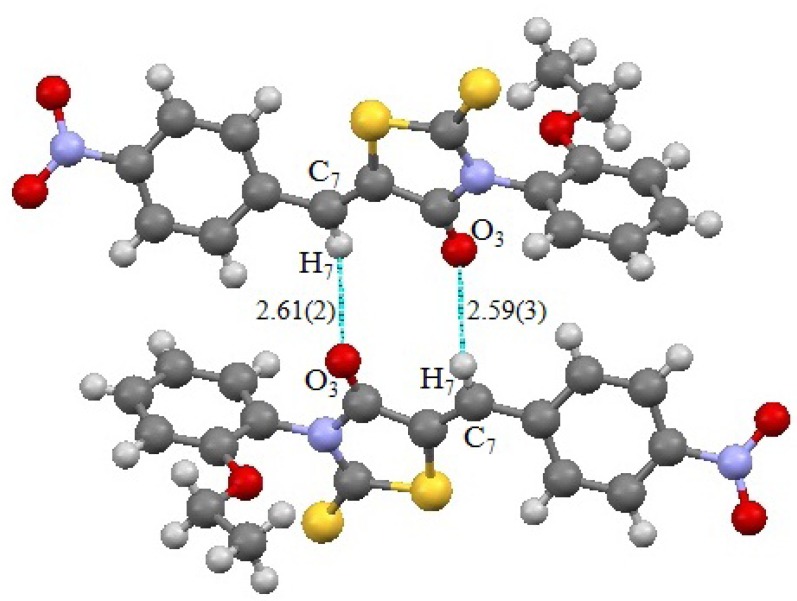
View of the two H-bonds C7-H7…O3 in the **2g** crystal.

The X-Ray diffraction structure of (*Z*)-**5**-(4-nitrobenzylidene)-3-*N*(2-ethoxyphenyl-2-thioxothiazolidin-4-one) is shown in Figure 5, with some hydrogen bonds. This compound crystallizes in the triclinic system.

X-Ray diffraction results agree with those given by the IR spectroscopy. On the other hand, the chirality of this type of compound is evidenced by the high value of the dihedral angle (79.7°) formed by the heterocyclic ring and the aryl linked at nitrogen in position 3 [15].

## 3. Experimental

### 3.1. General

All melting points were determined with a Büchi melting point apparatus (type Nr. 535) and are uncorrected. IR spectra were recorded as KBr pellets on a JASCO FT/IR4200 Fourier transform infrared spectrometer and the reported wavenumbers are given in cm^−1^. ^1^H-NMR spectra in CDCl_3_solution were recorded on a Bruker AC 250 instrument at 298 K. Chemical shifts are reported as δ (ppm) relative to TMS as internal standard. Coupling constants (*J*) are reported in Hz. The progress of the reactions was monitored by thin layer chromatography with silica-gel MERCK 60F_254_ (Merck, Darmstadt, Germany) using 8:2 dichloromethane-ethanol as eluent. Crystallographic data for the structure reported in this article have been deposited with the Cambridge Crystallographic Data Centre as supplementary publication number CCDC 805892. Copies of the data can be obtained free of charge on application to CCDC, 12 Union Road, Cambridge CB2 1EZ, UK. Fax: +44-1223-336-033; E-mail: deposit@ccdc.cam.ac.uk.

### 3.2. Typical Procedure for the Preparation of N-Arylthiazolidin-4ones *e, f*

DTC (0.2 mol, obtained from CS_2_ and the appropriate 2-alkyloxysubstituted aniline in 35% ammonia solution) was dissolved in ice cold water (300 mL). To this DTC solution was added sodium chloroacetate salt (0.22 mol) in two portions. After two hours of stirring, the mixture is acidified with concentrated HCl. The solid obtained is filtered, washed with water and then recrystallized from ethanol.

*2-Thioxo-3-N-(2-methoxyphenyl)thiazolidin-4-one* (**e**). Yield: 38%; white solid; mp: 142 °C; IR (cm^−^^1^): 1753 (C=O), 1257 (C=S); ^1^H-NMR: 3.80 (s, 3H), 4.180, 4.164 (AB, 2H, *J*_AB_ = 18.13), 7.45–7.01 (m, 4H); ^13^C-NMR: 36.27 (CH_2_), 55.84 (OCH_3_), 112.36, 121.22, 123.39, 131.47, 129.87, 154.17 (Aryl), 172.99 (C=S), 200.99 (C=O).

*2-Thioxo-3-N-(2-ethoxyphenyl) thiazolidin-4-one* (**f**). Yield 36%; white solid; mp: 118 °C; IR (cm^−^^1^): 1,742 (C=O), 1,223 (C=S); ^1^H-NMR: 1.33 (t, 3H, O-CH_2_-CH_3_, *J*^3^ = 6.95 Hz), 4.06 (oct, 1H, ABX_3_, *J*^2^ = 2.27, *J*^3^ = 6.95, O-CH_2_-CH_3_), 4.10 (oct, 1H, ABX3, *J*^2^ = 2.27, *J*^3^ = 6.95, -O-CH_2_-CH_3_), 4.18–4.18 (AB, 2H, *J*_AB_ = 18.11), 7.16–7.04 (m, 4H); ^13^C-NMR: 14.05 (O-CH_2_-CH_3_), 36.27 (CH_2_-S), 55.84 (O-CH_2_-CH_3_), 112.36, 121.02, 123.39, 129.87, 131.47, 154.17, 172.99 (C=S), 200.99 (C=O).

### 3.3. Typical Procedure for the Preparation of 5-Arylidenethiazolidin-4-ones *1g–10g*

To a solution of equimolar amounts of 3-*N*(2-alkyloxyaryl)-2-thioxothiazolidinone (10^−2^ mol) and the appropriate aromatic aldehyde in glacial acetic acid (30 mL), was added sodium acetate (0.015 mol). The mixtures were then refluxed for different periods (2–7 h), monitored by thin-layer chromatography. The reaction mixture was cooled and poured onto crushed ice. The yellow solids obtained were filtered, washed with water and recrystallized from a suitable solvent. Ten compounds **1g–10g** were synthesized by adopting the same procedure. 

*(*Z*)-5-(4-Chlorobenzylidene)-3-*N*(2-ethoxyphenyl)-2-thioxothiazolidin-4-one* (**1g**) [13]. Yield: 95%; mp: 170 °C; IR (cm^−^^1^): 3,494 broad band, 3,027 (C-N), 1,707 (C=O), 1,256 (C=S); ^1^H-NMR: 1.31 (t, 3H, -O-CH_2_-CH_3_, *J* = 6.95), 4.06 (oct, 1H, ABX_3_, *J*^2^ = 2.2, *J*^3^ = 6.95) (-O-CH_2_-CH_3_), 4.10 (oct, 1H, ABX_3_, *J*^2^ = 2.2, *J*^3^ = 6.95, -O-CH_2_-CH_3_), 7.23–7.09 (AB, 4H aryl, *J* = 7.21), 7.44–7.29 (m, 4H), 7.73 (s, 1H, -CH=C-); ^13^C-NMR: 14.66 (O-CH_2_-CH_3_), 64.42 (-O-CH_2_-CH_3_), 113.47, 120.95, 123.62, 124.33, 129.56, 129.68, 129.84, 131.26, 131.35, 131.44, 131.68, 131.95, 136.80, 154.28, 167.19 (C=S), 192.65 (C=O). 

*(*Z*)-5-(4-Nitrobenzylidene)-3-*N*(2-ethoxyphenyl)-2-thioxothiazolidin-4-one* (**2g**) [15]. Yield: 75%; mp: 210 °C; IR (cm^−^^1^): 3,407 broad band, 3,035 (C-N-CS), 1,710 (C=O), 1,256 (C=S); ^1^H-NMR: 1.42 (t, 3H, -O-CH_2_-CH_3_, *J*^3^ = 6.97), 4.06 (oct, 1H, ABX_3_, *J*^2^ = 2.2, *J*^3^ = 6.95, -O-CH_2_-CH_3_), 4.11 (oct, 1H, ABX_3_, *J*^2^ = 2.2; *J*^3^ = 6.95, -O-CH_2_-CH_3_), 7.53–7.08 (m, 4H), 7.71 (d, 2H, *J* = 8.75) 7.79 (s, 1H, -CH=C-), 8.53 (d, 2H, *J* = 8.80); ^13^C-NMR: 14.17 (O-CH_2_-CH_3_), 64.43(-O-CH_2_-CH_3_), 113.48, 121.00, 123.26, 124.46, 128.35, 129.12, 129.77, 130.95, 131.63, 139.44, 147.96, 154.21, 167.19 (C=S), 191.65 (C=O). 

*(*Z*)-5-(4-(2-Nitrophenyl)butadienylene)-3-*N*(2-methoxyphenyl)-2-thioxothiazolidin-4-one* (**3g**). Yield: 90%; mp: 233 °C; IR (cm^−^^1^): 3,423 (broad band), 3,035 (C-N-CS), 1,716 (C=O), 1,238 (C=S); ^1^H-NMR: 3.80 (3H, s), 6.75 (1H, m), 7.05–7.20 (3H, m), 7.46–7.75 (6H, m), 8.03 (1H, CH=C,* J* = 11.54); ^13^C-NMR: 55.92 (O-CH_3_), 112.46, 121.05, 123.38, 125.02, 125.19, 127.40, 128.25, 169.71, 129.10, 130.87, 131.22, 131.54, 133.48, 138.12, 148.03, 154.02, 166.04 (C=S), 191.90 (C=O).

*(*Z*)-5-(3,4-Dimethylbenzylidene)-3-*N*(2-ethoxyphenyl)-2-thioxothiazolidin-4-*one (**4g**). Yield: 82%; mp: 136 °C; IR (cm^−^^1^): 3,434 (broad band), 3,025 (C-N-CS), 1,725 (C=O), 1,239 (C=S); ^1^H-NMR: 1.31 (3H, t, *J*^3^ = 6.89,O-CH_2_-CH_3_), 2.38 (3H, s, -CH_3_), 2.49(3H, s, -CH_3_), 4.05 (oct, 1H, ABX_3_, *J*^2^ = 2.35; *J*^3^ = 6.89) (-O-CH_2_-CH_3_),4.07 (oct, 1H, ABX_3_, *J*^2^ = 2.38, *J*^3^ = 6.89, -O-CH_2_-CH_3_); 7.04–7.48 (7H, H arom.), 7.97 (1H, s, HC=C_het_); ^13^C-NMR: 14.69 (CH_3_), 19.50 (CH_3_), 21.07 (O-CH_2_-CH_3_), 64.46 (O-CH2), 113.51 (C_het_), 120.94, 123.89, 124.48, 128.61, 129.87, 131.10, 131.33, 131.52, 132.33, 136.31, 136.53, 154.36, 157.14 (C=S), 194.09 (C=O).

*(*Z*)-5-(3-Methoxy-4-hydroxybenzylidene)-3-*N*(2-ethoxyphenyl)-2-thioxothiazolidin-4-one* (**5g**). Yield: 75%; mp: 144 °C; IR (cm^−^^1^): 3,419 (broad band), 3,027 (C-N-CS), 1,712 (C=O), 1,249 (C=S); ^1^H-NMR: 1.29 (3H, *J*^3^ = 6.79, t-O-CH_2_-CH_3_), 3.98 (3H, s O-CH_3_), 4.04 (oct, 1H, ABX_3_, *J*^2^ = 2.42, *J*^3^ = 6.79, -O-CH_2_-CH_3_), 4.07 (oct, 1H, ABX_3_, *J*^2^ = 2.34, *J*^3^ = 6.79, -O-CH_2_-CH_3_), 6.04 (1H, s broad -OH), 7.01–7.48 (m, 7H arom.), 7.71 (1H, s, HC=C); ^13^C-NMR: 14.68, 56.11, 64.44, 112.09, 113.51, 115.39, 120.53, 120.94, 123.92, 125.11, 126.21, 129.89, 131.31, 133.42, 147.08, 148.46, 154.35, 167.42, 192 (C=S), 193 (C=O).

*(*Z*)-5-(Furan-2-ylmethylene)-3-*N*(2-ethoxyphenyl)-2-thioxothiazolidin-4-one* (**6g**). Yield: 53%; mp: 140 °C, IR (cm^−^^1^): 3,414 (broad band), 3,065 (C-N-CS), 1,716 (C=O), 1,227 (C=S); ^1^H-NMR: 1.28 (t, 3H, *J*^3^ = 6.97), 4.05 (oct, 1H, ABX_3_, *J*^2^ = 2.39, *J*^3^ = 6.57) (-O-CH2-CH3), 4.07 (oct, 1H, ABX_3_, *J*^2^ = 2.35, *J*^3^ = 6.62, -O-CH2-CH3), 6.04 (m, 1H), 6.84 (d, 1H), 7.06 (m, 2H), 7.20 (m, 2H), 7.44 (m, 1H), 7.51 (s, 1H, HC=C); ^13^C-NMR: 14.66, 64.44, 113.49 (2C), 118.20, 118.40, 120.90, 121.69, 123.95, 129.93, 131.29, 146.92, 150.26, 154.37, 167.11 (C=S), 194.11 (C=O).

*(*Z*)-5-(4-Hydroxybenzylidene)-3-*N*-(2-methoxyphenyl)-2-thioxothiazolidin-4-one* (**7g**). Yield: 73%; mp: 235 °C; IR (cm^−^^1^): 3,350 (broad band), 3,065 (C-N-CS), 1,694 (C=O), 1,229 (C=S); ^1^H-NMR: 3.74 (s, 3H), 5.74 (s broad, 1H), 6.95 (m, 8H), 7.75 (s, 1H, HC=C); ^13^C-NMR: 55.88 (-O-CH_2_-CH_3_), 112.66 (2C), 116.66, 118.09, 120.82, 123.36, 123.94, 130.14, 131.32, 133.34 (2C), 133.93, 154.62, 160.72, 166.55 (C=S), 193.16 (C=O).

*(*Z*)-5-(4-Hydroxybenzylidene)-3-*N*(2-ethoxyphenyl)-2-thioxothiazolidin-4-one* (**8g**). Yield: 80%; mp: 223 °C; IR (cm^−^^1^): 3,361 (broad band), 3,150 (C-N-CS), 1,702 C=O, 1,216 C=S; ^1^H-NMR: 1.16 (t, 3H, *J*^3^ = 6.94), 4.04 (oct, 1H, ABX_3_, *J*^2^ = 2.42, *J*^3^ = 6.63) (-O-CH_2_-CH_3_), 4.06 (oct, 1H, ABX_3_, *J*^2^ = 2.82, *J*^3^ = 5.66, -O-CH_2_-CH_3_), 5.74 (s, 1H), 6.93–7.57 (m, 8H), 7.79 (s, 1H CH=C); ^13^C-NMR: 14.43 (OCH_2_CH_3_), 64.01 (OCH_2_CH_3_), 113.79 (2C), 116.70, 117.93, 120.79, 121.09, 123.62, 130.12, 131.19, 133.37, 133.76 (2C), 153.66, 160.69, 166.16 (C=S), 193.13.

*(*Z*)-5-(3-Hydroxybenzylidene)-3-*N*(2ethoxyphenyl)-2-thioxothiazolidin-4-one* (**9g**). Yield: 35%; mp: 180 °C; IR (cm^−^^1^): 3,406 (broad, OH); 2,973.7 (C-H_arom_.); 2,973.7 (C-H aliph.); 1,692 (C=O); 1,601–1,557.24 (C=C); 1,358.60 (CH3); 1,24O (C=S); 750.17–810.92 arom. subt.); ^1^H-NMR: 1.16 (t, 3H); 4.04(oct, 1H, ABX_3_, -O-CH2-CH3); 4.06 (oct, 1H, ABX_3_, -O-CH2-CH3); 5.74 (s, 1H), 6.91–6.95 (m, 1H), 7.71 (s, 1H CH=C), 7.01–7.14 (m, 3H); 7.19–7.26 (m, 2H), 7.34–7.40 (m, 1H), 7.43–7.49 (m, 1H); ^13^C-NMR: 14.64 (OCH_2_CH_3_), 64.09 (OCH_2_CH_3_), 113.48, 117.56, 118.36, 120.90, 121.30, 121.87, 123.25, 124.56, 129.77, 130.63, 131.98, 132.98, 152.42, 160.67, 168.89 (C=S), 193.00 (C=O).

*(*Z*)-5-(4-Cyanobenzylidene),3-*N*(2-ethoxyphenyl)-2-thioxothiazolidin-4-one* (**10g**). Yield: 62%; mp: 213 °C; IR (cm^−^^1^): 3,417 (broad, weak intensity, OH); 3,061(C-N-CS); 2,978.52 (C-H_arom_.); 2,904.27 (C-H_aliph_.); 2,222 (CN); 1,718.26 (C=O); 1,598.70–1,496.49 (C=C); 1,359.57 (CH_3_); 1,257.36 (C=S); 759.81–823.45 (subst. arom.); ^1^H-NMR: 1.29 (t, 3H, *J* = 6.95); 4.04 (oct, 1H, ABX_3_, *J*^2^ = 2.91; *J*^3^ = 6.91,-O-CH2-CH3); 4.09 (oct, 1H, ABX_3_, *J*^2^ = 2.91, *J*^3^ = 6.91, -O-CH2-CH3); 7.06–7.12 (m, 2H); 7.18–7.22 (m, 1H); 7.45–7.51 (m, 1H); 7.45–7.51 (m, 1H); 7.63 (d, 2H, AB, *J* = 7.58); 7.75 (s, 1H, CH=C); 7.78 (2H, AB, *J* = 7.58); ^13^C-NMR: 14.64 (OCH_2_CH_3_), 62.62 (OCH_2_CH_3_), 113.84 (2C), 117.29, 118.84, 120.81, 121.56, 122.34, 130.63, 131.58, 132.98, 136.94 (2C), 142.23, 152.42, 160.29, 193.17 (C=S), 200.57 (C=O).

## 4. Conclusions

In summary, ten 5-arylidene-2-thioxo-3-*N*-arylthiazolidin-4-ones were synthesized and isolated in good yields. The reaction seems to be dependent on the electron-withdrawing or electron-donating character of substituents present in the aldehydes. Structure determination was accomplished by IR, NMR and X-Ray diffraction. These chiral molecules have a strong electronic delocalization. This is highlighted by the variation of the wavelength of the carbonyl function depending on the nature of the arylidene moiety. These compounds appear to be potentially good candidates for application in the field of the solar cells and nonlinear optics.
